# SDGTrack: A Multi-Target Tracking Method for Pigs in Multiple Farming Scenarios

**DOI:** 10.3390/ani15111543

**Published:** 2025-05-24

**Authors:** Tao Liu, Dengfei Jie, Junwei Zhuang, Dehui Zhang, Jincheng He

**Affiliations:** College of Mechanical and Electrical Engineering, Fujian Agriculture and Forestry University, Fuzhou 350002, China

**Keywords:** computer vision, multi-object tracking, multi-scene generalization, group-housed pigs

## Abstract

In pig farming, multi-object tracking (MOT) algorithms are effective tools for identifying individual pigs and monitoring their health, which enhances management efficiency and intelligence. However, due to the considerable variation in breeding environments across different pig farms, existing models often struggle to perform well in unfamiliar settings. To enhance the model’s generalization in diverse tracking scenarios, we have innovatively proposed the SDGTrack method. This method improves tracking performance across various farming environments by enhancing the model’s adaptability to different domains and integrating an optimized tracking strategy, significantly increasing the generalization of group pig tracking technology across different scenarios. To comprehensively evaluate the potential of the SDGTrack method, we constructed a multi-scenario dataset that includes both public and private data, spanning ten distinct pig farming environments. We only used a portion of the daytime scenes as the training set, while the remaining daytime and nighttime scenes were used as the validation set for evaluation. The experimental results demonstrate that SDGTrack achieved a MOTA score of 80.9%, an IDSW of 24, and an IDF1 score of 85.1% across various scenarios. Compared to the original CSTrack method, SDGTrack improved the MOTA and IDF1 scores by 16.7% and 33.3%, respectively, while significantly reducing the number of ID switches by 94.6%. These findings indicate that SDGTrack offers robust tracking capabilities in previously unseen farming environments, providing a strong technical foundation for monitoring pigs in different settings.

## Simple Summary

This study presents a novel method for the effective detection and tracking of pigs in unknown environments and complex scenarios. Using the CSTrack model as the baseline, we enhance it by incorporating an environment-aware adaptive module and optimizing the target association strategy to address the model’s limited tracking ability in unknown scenes. Experimental results show that, compared to several advanced models, this method performs excellently in various complex environments, meeting the tracking requirements in unknown scenarios and providing robust technical support for the precise management of pigs.

## 1. Introduction

With population and economic growth, the demand for pork continues to rise [1], presenting new opportunities and challenges for the swine industry. Rising labor costs and inefficiencies in pig farming highlight the need for intelligent farming practices, making continuous tracking and monitoring of pigs in complex environments increasingly important [2]. Although recent advances in computer vision have contributed to pig monitoring, most existing methods are validated only in controlled or idealized scenarios, failing to reflect the complexity of real-world farming conditions. In practical applications, environmental variations often reduce detection accuracy and cause frequent target loss, severely limiting the generalization and robustness of current models [3]. Therefore, it is essential to develop a target tracking model with strong cross-scenario adaptability to ensure reliable identification and stable performance under dynamic farming conditions.

Early methods for pig identification and positioning primarily relied on Radio Frequency Identification (RFID) technology, using electronic ear tags for automated tracking within a reader’s range [4]. This approach effectively managed individual pigs, particularly in tracking feeding and drinking behaviors. UHF-RFID technology was later applied to monitor feeder visits in growing-finishing pigs, underscoring its potential for animal behavior monitoring [5]. Subsequent advancements involved High-Frequency RFID (HF RFID) systems that used advanced multiplexers to connect multiple antennas to a single reader, enabling the precise monitoring of multiple pigs simultaneously [6,7]. Additionally, an RFID-based real-time alert system was developed to detect potential health issues by tracking individual pigs’ feeding and drinking behaviors, demonstrating high specificity in behavior identification [8]. However, the limitations of RFID technology have become increasingly apparent over time. High equipment and maintenance costs have become a heavy burden for long-term applications. Additionally, the technology may induce stress responses in pigs [2], complicating management and indirectly affecting data completeness and analytical accuracy.

With the development of deep learning, video-based multi-object tracking (MOT) methods like Tracking-by-Detection (TBD) and Joint Detection and Embedding (JDE) have become increasingly prominent [9]. TBD methods, which separate detection and association tasks, have been applied in pig farming to improve tracking accuracy and stability. Enhancements to two-stage trackers, like Faster R-CNN [10] and Mask R-CNN [11], have improved mask fitting and occlusion handling in pig tracking applications [12,13]. However, due to the limited speed of two-stage detectors, single-stage detectors have gained popularity for their efficiency. In complex environments, the optimized YOLOv5-Byte method integrates behavioral information and ID values to enhance detection accuracy and maintain continuous tracking, while effectively reducing ID association errors [14]. Similarly, using YOLOX-S and YOLOv5s as detectors has resulted in improved MOTA and IDF1 scores and a substantial reduction in ID switches [15]. For complex tracking scenarios, an MPC-YD method based on YOLOv5 was proposed, achieving high detection precision and reliable multi-object tracking performance [16].

Compared to two-stage tracking methods, the Joint Detection and Embedding (JDE) approach [17] integrates detection and re-identification tasks into a single framework. This integration eliminates information gaps between detection and tracking found in traditional methods and streamlines the workflow by reducing redundant computations. Consequently, JDE significantly enhances processing efficiency while maintaining high accuracy, making it well-suited for real-time multi-object tracking. Building on Joint Detection and Embedding algorithms, Guo et al. proposed a weighted association algorithm that improves tracking performance [17]. Their use of FairMOT combined with the weighted association algorithm achieved optimal tracking performance for pigs, with IDF1 and MOTA scores reaching 90.3% and 90.8%, respectively. Guo et al. compared three state-of-the-art deep learning-based MOT methods [18]: Joint Detection and Embedding (JDE), FairMOT, and YOLOv5s with DeepSORT [19]. By incorporating the improved weighted association method, they analyzed the continuous monitoring capabilities of each model for pig tracking. The results showed that the FairMOT method with the suggested weighted association achieved the highest IDF1 score, the fewest ID switches, and the fastest execution rate.

While existing research has made significant progress in tracking within specific scenarios, the performance of these models often drops sharply when applied to new or unknown environments due to their limited generalization capabilities. This is primarily because most models rely heavily on background information specific to the training scenarios, lacking adaptability to diverse environments. In pig farming, scene diversity manifests in various aspects, such as lighting conditions and scene changes [20]. These factors pose significant challenges for models handling cross-scene tasks, as changes in background information can drastically affect identification and tracking performance in different farming environments. To address the issue of poor model performance in unknown scenarios, we have innovatively proposed SDGTrack, which introduces adaptive modules and improves existing tracking strategies. This enables the model to distinguish and learn background information across different scenes. Experimental results show that SDGTrack not only performs well in known scenarios but also maintains high tracking accuracy in unknown environments.

The key contributions of this work are threefold:

(1) An environment-aware adaptive module was proposed to enhance the model’s performance across various scenarios.
(2) A target association strategy was designed to effectively reduce target mismatches and misassignments.
(3) Comparative experiments with other leading trackers on MOT data across multiple scenarios validated the ability of SDGTrack to extend from a single environment to multiple scenarios.

## 2. Materials and Methods

### 2.1. Materials

#### 2.1.1. Data Acquisition

The experimental data comprises two main components. The first part consists of data collected by our team using a Xiaomi CW400 high-definition surveillance camera (4 megapixels, resolution: 2560 × 1440; manufactured by Xiaomi Corporation, Beijing, China). Recordings were conducted on 5 and 17 November 2023, at a pig housing unit in the experimental garden of Fujian Agriculture and Forestry University. Each pen within the pig house measured 2.6 m × 1.6 m and was equipped with a feeding trough, exhaust fan, surveillance system, and bowl-type drinker. Two three-way crossbred pigs were housed per pen, one white and one dark-colored, each weighing approximately 50 kg, were housed per pen. The camera was mounted on the ceiling at a height of 2.2 m, providing a vertical top-down view that fully covered the pen area. The camera was configured for continuous recording throughout the day; when ambient light levels fell below 5 lux, infrared illumination was automatically enabled.

To enhance environmental diversity and evaluate the model’s generalization ability, we additionally incorporated several publicly available datasets and privately collected video sequences [21,22,23,24], these supplemental datasets were captured under various farming conditions, covering both daytime and nighttime illumination.

#### 2.1.2. Dataset Construction

To support model training and evaluation, we constructed a multi-scenario pig tracking dataset by combining publicly available datasets and privately collected video resources. The dataset comprises ten distinct video sequences, including eight daytime scenes and two nighttime scenes, covering a variety of pig farming environments. The dataset is publicly available at https://github.com/hurry-baby/SDGTrack (accessed on 18 May 2025).

All ten videos were first preprocessed and standardized to a frame rate of 30 frames per second. From each video, we extracted a 1 min segment and subsequently annotated them following the Multiple Object Tracking (MOT) format [25]. The videos were then exported into frame-level image sequences, resulting in a total of 18,000 annotated images.

**Figure 1:**
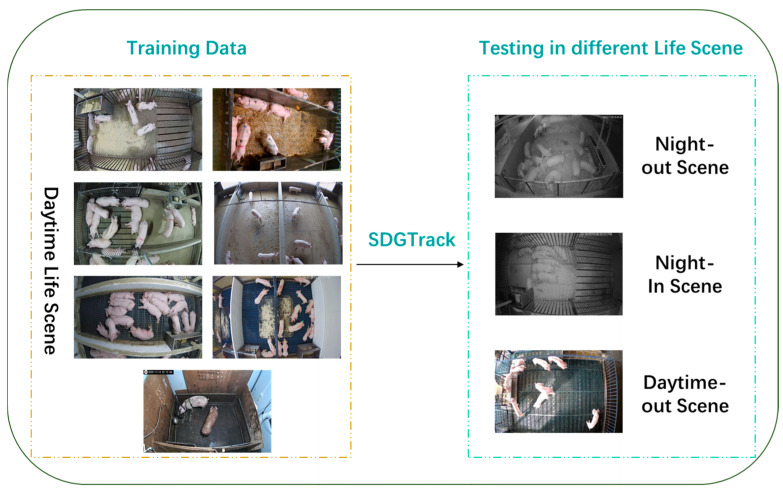
Diagram of the multi-scenario pig dataset. This dataset contains a total of ten pig farming scenarios, including eight daytime and two nighttime scenes. The goal of SDGTrack is to train the model on daytime scenarios so that it can generalize to other daytime and nighttime environments.

From the full dataset, we selected seven daytime sequences representing different farm settings as the training set, yielding 12,600 images reflecting diverse pig activity patterns under various daytime conditions. The remaining three sequences, which were not included in training, were designated as the test set. This test set includes one additional daytime scenario not seen during training (Daytime-Out Scene), and two nighttime scenarios: one representing the night version of an environment present in the training set (Night-In scene), and another from an entirely different pig house (Night-Out scene), used to evaluate the model’s generalization ability under both lighting and environmental changes. Specific information on the composition of the data is shown in Table 1:

The Night-In Scene tests the model’s performance under changing lighting conditions. Although the model has been trained in the same environment during the day, the nighttime scenes present significant changes in lighting and background information. The Night-Out scene evaluates the model’s generalization to a completely unfamiliar night environment. This test is more stringent than the first, as the model must not only adapt to lighting changes but also to a completely different farm environment. Finally, the daytime scenes from a different farm assess the model’s performance in unfamiliar daytime conditions. Although the lighting is similar to the training set, the differing farm layouts are likely to impact the model’s performance. As shown in Figure 1, the three test scenarios represent distinct environmental changes to evaluate the model’s tracking performance in unknown or changing environments.

### 2.2. Methods

#### 2.2.1. Basic JDE and CSTrack Methods

The Joint Detection and Embedding (JDE) model [26] effectively integrates object detection and identity re-identification (re-ID) into a single network architecture. By utilizing joint learning, the JDE model not only accurately localizes objects but also associates identity information across consecutive frames, significantly improving tracking efficiency and reducing computational complexity. The baseline network of JDE is derived from YOLOv3’s [27] Darknet-53 and Feature Pyramid Network (FPN).The CSTrack model [28] builds on JDE by further optimizing both detection and re-ID tasks. It addresses the issue of “over-competition” between these tasks, where competition in representation learning can cause confusion and negatively impact overall performance. Architecturally, CSTrack enhances JDE by incorporating a feature decoupling module, creating separate feature maps tailored to each task, allowing detection and re-ID to learn independently and improve performance.

For detection, CSTrack replaces the original YOLOv3 framework with the faster and more advanced YOLOv5. Additionally, it introduces a scale-aware attention network that leverages spatial and channel attention to capture appearance information across different scales, optimizing re-ID feature representation and further enhancing re-ID performance.

#### 2.2.2. SDGTrack Tracking Model

**Figure 2:**
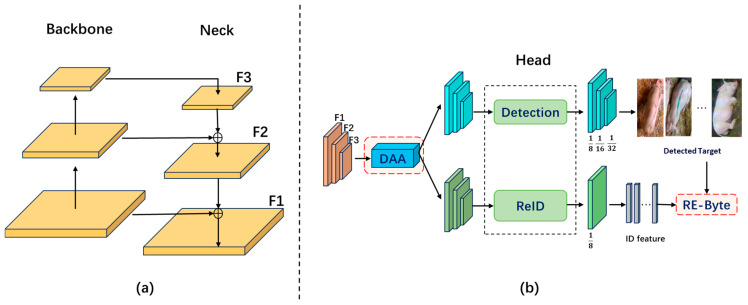
SDGTrack. (**a**) is the feature extractor including backbone and neck; (**b**) illustrates our proposed prediction structure of SDGTrack.

To address the limitations of traditional models in tracking pigs across different farm environments, this study proposes SDGTrack, a multi-object tracking solution that employs single-domain adaptive generalization. The framework is built upon CSTrack [28], as illustrated in Figure 2, where (a) shows the feature extraction structure, including the Backbone and Neck components, and (b) presents the prediction module proposed in this study. To reduce performance discrepancies across environments, we first introduce an enhanced domain-aware attention (DAA) module. This module learns feature information from multiple environments, enabling the model to selectively emphasize relevant background features and suppress less useful ones, thereby achieving adaptive representation for unseen domains.

Additionally, acknowledging the reduced reliability of re-identification (Re-ID) features in varying validation environments, we proposed the Re-Byte approach, building upon ByteTrack [29]. By splitting the matching task into high- and low-confidence categories, the model enhances its focus on low-confidence detections while efficiently completing the association process through a combination of IoU (Intersection over Union) distance and identity feature (ID feature) distance.

SDGTrack learns the three tasks of classification, bounding box regression, and appearance features in parallel, like the JDE [26] model. The total loss function is defined as Equation (1).

(1) $$L_{total}=\sum_{i}^{M}\sum_{j=\alpha ,\beta ,\gamma }\frac{1}{2}(\frac{1}{e^{s_{j}^{i}}}L_{j}^{i}+s_{j}^{i})$$

where the loss function consists of three components: the classification loss $L_{\alpha }$, the bounding box regression loss $L_{\beta }$, and the embedded learning loss $L_{\gamma }$. $s_{j}^{i}$ stands for task-specific-independent uncertainty and is a tunable network parameter. M is the number of predictor heads.

#### 2.2.3. Domain-Aware Attention Module

**Figure 3:**
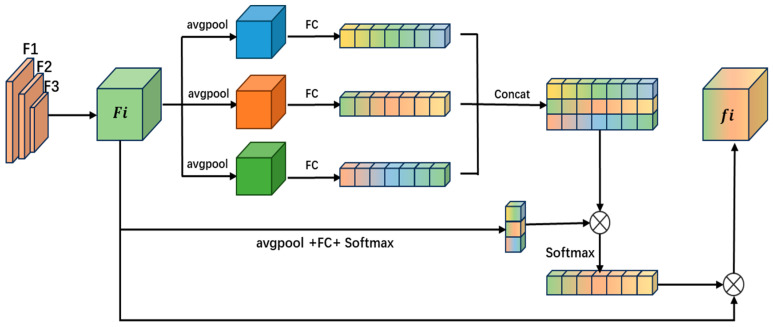
DAA module.

To enable the model to adaptively learn shared feature information across different scenarios, we introduce an improved domain-aware attention module. This module achieves this by adaptively weighting multiple Effective Squeeze-and-Excitation (ESE) blocks [30]. The architecture is illustrated in Figure 3. The domain-aware attention module first performs global pooling on the input feature map to aggregate spatial information and generate a global feature vector. Then, global pooling and fully connected operations are applied, followed by another fully connected layer and a softmax function to produce domain-sensitive weights. These weights are used to adaptively weight the domain-related features generated by the three ESE modules, as shown in the following equation:

(2) $$S_{DDA}=\mathrm{softmax}(W_{C}F_{a\nu g}(F_{i}))$$

where $W_{C}$ is the fully connected weight matrix, $F_{a\nu g}$ is the global average pooling operation, and the softmax function ensures that the sum of the elements in the generated weight vector $S_{DDA}$ is 1, so that it can be used for weighting and combining. The generated domain-sensitive weight vector $S_{DDA}$ is used to weight the output $S_{C}$ produced by the splicing of the three ESE modules to form a domain-adaptive response vector $S_{DC}$, which is computed as follows:

(3) $$S_{DC}=S_{DDA}S_{C}$$

Next, the domain adaptive response vector $S_{DC}$ is used to adaptively weight the channels of the original input feature map $F_{i}$, and the model’s perception of the environment is realized by converting $S_{DDA}$ to the scaling factor of the input features of $F_{i}$ through the sigmoid function. The calculation formula is as follows:

(4) $$f_{i}=F_{scale}(F_{i},\sigma (S_{DC}))$$

where $F_{scale}(\cdot )$ is a multiplication operation performed on the channels of the feature map and σ is a sigmoid function.

This approach, unlike the hard attention mechanism, allows cross-domain information sharing and improves the effectiveness of feature representation. With a domain-aware attention network, the model can better adapt to different environments and achieve robustness and accuracy for multi-target tracking tasks.

#### 2.2.4. Re-Byte

**Figure 4:**
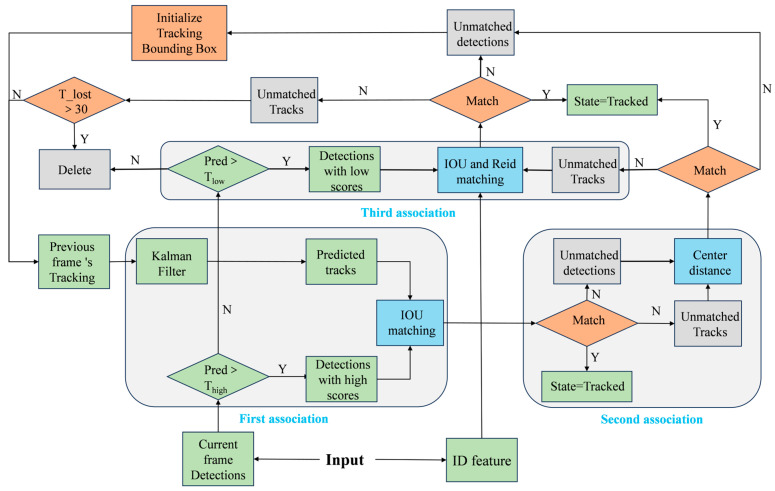
Re-Byte tracking flow chart.

To improve the model’s generalization ability in dynamic scenarios, we optimized the matching strategy in the Multiple Object Tracking (MOT) algorithm. Traditional tracking methods often rely on high-confidence detection boxes. However, in changing or unknown environments, detection accuracy may vary significantly, resulting in poor target matching when accuracy is affected by scene changes. Inspired by ByteTrack, we proposed the Re-Byte method. By dividing detection boxes into high and low thresholds, we enrich the target matching information, reducing the negative impact of scene changes, lighting variations, and occlusions on subsequent tracking tasks. This enhances the model’s generalization across complex conditions. Figure 4 illustrates the improved Re-Byte tracking process, which integrates multiple factors for target matching, significantly enhancing the model’s adaptability and robustness in challenging environments.

In the initial matching stage, we first screened all candidate detection boxes and categorized them into high- and low-threshold groups. The primary focus of this stage is to match high-threshold detection boxes with the currently tracked targets. We used a Kalman filter [31] to predict the potential positions and trajectories of all tracked targets in the current frame. The IoU distance [32] between the detection boxes and the predicted trajectories was then calculated. Finally, the Hungarian algorithm [33] was applied to achieve optimal matching between the detection boxes and the predicted trajectories. The IoU calculation formula is shown in Equation (5):

(5) $$IoU=\frac{A\cap B}{A\cup B}$$

where A denotes the detected target frame, B denotes the predicted frame obtained from the tracked target using the Kalman filtering method, and the value domain of IoU is [0, 1].

Unlike ByteTrack, we introduce an additional matching step in the tracking process to enhance performance. In this second matching phase, we re-associate unmatched detection boxes with predicted tracks. This association is based on the Euclidean distance [34] to evaluate the similarity between them. The goal is to address cases where high-confidence targets are not well-associated due to low overlap between bounding boxes caused by occlusion or other factors. The formula for calculating the Euclidean distance is shown in Equation (6).

(6) $$d_{\mathrm{track}}=\sqrt{{\left(x_{\mathrm{pred}}-x_{\det}\right)}^{2}+{\left(y_{\mathrm{pred}}-y_{\det}\right)}^{2}}$$

where $\left(x_{\det},y_{\det}\right)$ is the center point coordinate of the detection frame, and $\left(x_{\mathrm{pred}},y_{\mathrm{pred}}\right)$ is the center point coordinate of the predicted trajectory.

For tracks that remain unmatched after the first two steps, we apply a third association using low-threshold detection frames. In this step, we combine the IOU distance and embedded feature distance through weighted fusion, with the latter calculated using cosine similarity. By considering both feature distances together, the model effectively leverages the inherent characteristics of targets, enhancing its performance in multi-object tracking tasks across various scenarios.

#### 2.2.5. Evaluation Metrics

To comprehensively analyze the tracking accuracy of our model across different scenarios, we utilized the metrics derived from the MOT challenge based on pedestrian datasets [25]. Additionally, we incorporated the evaluation metrics used in the JDE [26] and FairMOT [35] methods to assess our proposed approach alongside other methods. The key metrics include MOTA, IDF1, MOTP, HOTA, MT, ML, IDS, FP, FN, and FPS. Among these, Multi-Object Tracking Accuracy (MOTA) is considered the most critical metric in multi-object tracking, as it evaluates the overall accuracy of a multi-object tracking algorithm. The calculation formula for MOTA is provided below.

(7) $$MOTA=1-\frac{FN+FP+IDSW}{GT}$$

where GT represents the number of ground truth objects. The maximum value of MOTA is 1, while its minimum value can reach negative infinity. FN and FP denote the numbers of missed detections and false positives, respectively. IDSW counts the instances of ID switches, indicating how often the tracker incorrectly assigns or changes object IDs. The smaller the IDSW, the better the tracker maintains object identity. IDF1 measures the tracker’s consistency in preserving object identity by combining ID precision and ID recall. The formula for calculating IDF1 is as follows:

(8) $$IDF1=\frac{2IDTP}{2IDTP+IDFP+IDFN}$$

where IDTP represents the number of correctly matched objects, IDFP denotes the number of incorrectly matched objects, and IDFN refers to the number of missed objects. Multiple Object Tracking Precision (MOTP) is a metric used to measure the positional error in tracking, and its expression is as follows:

(9) $$MOTP=\frac{\sum_{t,i}d_{t.i}}{\sum_{t}c_{t}}$$

where $c_{t}$ represents the number of detection boxes that successfully match with ground truth in frame t, while $d_{t.i}$ measures the distance between matched pairs. Higher Order Tracking Accuracy (HOTA) is a unified metric that jointly evaluates detection and association performance across multiple IoU thresholds. It is computed as follows:

(10) $$HOTA=\frac{1}{|A|}\underset{\alpha \in A}{\sum }{Det}_{\alpha }\cdot {AssA}_{\alpha }$$

where A={0.05,0.10,…, 0.95} denotes a set of fixed IoU thresholds, and ${\mathrm{Det}}_{\alpha }$, ${\mathrm{AssA}}_{\alpha }$ denote the detection and association accuracy at threshold α, respectively.

All evaluation metrics used in this study are summarized in Table 2. The goal is to provide a comprehensive and detailed assessment of the model’s tracking performance in complex scenarios. The table lists various terms used to evaluate tracking accuracy and clearly indicates the ideal trends for each metric. An upward arrow next to a metric name signifies that higher values correspond to better model performance, while a downward arrow indicates that lower values are preferable.

## 3. Results and Analysis

### 3.1. Experimental Platforms

We use an NVIDIA GeForce RTX 4090 chip with 24 GB of RAM (NVIDIA, Santa Clara, CA, USA) as the graphics card for core computation. The CPU model is Intel Core i7-13700X, 3.40 GHz. The version of the CUDA compiler is 11.8, the version of Python is 3.8.18, and the version of PyTorch used in the work is 2.1.1. All experiments were performed on this device.

### 3.2. Comparative Experiments with Different MOT Algorithms

In order to evaluate the effectiveness of our proposed SDGTrack approach, we compared it to several state-of-the-art multi-target tracking benchmark models. To ensure the fairness and consistency of the comparison, we matched all the model configurations involved in the comparison to those in the CSTrack model, which mainly include the model size and the specific parameters of the data enhancement strategy. The data are all in our home-grown dataset, as shown in Figure 1.

The comparison results of each model are shown in Table 3, and our proposed SDGTrack model demonstrates outstanding performance across the majority of evaluation metrics. In particular, it achieves a MOTA of 80.9%, indicating strong overall tracking accuracy by minimizing false positives, false negatives, and identity switches. The model also attains an IDF1 score of 85.1%, reflecting its excellent ability to maintain consistent object identities throughout the tracking sequence. Furthermore, SDGTrack significantly reduces identity switches, with a total of only 24, representing a 94.6% reduction compared to the baseline. This reduction highlights the model’s robustness in preserving target identities across frames. In addition, SDGTrack achieves a HOTA score of 83.0%, which demonstrates its balanced performance in both spatial detection accuracy and temporal identity association. Although FairMOT [35] achieved the highest frame rate at 40.5 FPS, its MOTA and IDF1 scores were considerably lower (55.6% and 54.6%, respectively), indicating weaker tracking stability and accuracy. In comparison, our proposed SDGTrack achieved a competitive speed of 27.5 FPS while delivering significantly better accuracy. Overall, SDGTrack consistently outperformed other models across all evaluation scenarios.

In addition to evaluating the overall performance of the models, we also analyzed the metrics of the top four models in three different scenarios to understand their precision advantages and characteristics. As shown in Table 4, in the nighttime scenario within the daytime training dataset, SDGTrack achieved a MODA of 78.1%, an IDF1 of 81.9%, and a HOTA of 80.0%. In comparison, the best-performing baseline model, FairMOT, obtained a MODA of 68.8%, an IDF1 of 56.8%, and a HOTA of 62.5%, with our model surpassing it by 9.3%, 25.1%, and 17.5%, respectively. In the nighttime scenario without the daytime training dataset, SDGTrack achieved a MODA of 69.6%, an IDF1 of 82.6%, and a HOTA of 75.8%. In contrast, the best-performing baseline model, CSTrack, achieved a MODA of 52.8%, an IDF1 of 44.4%, and a HOTA of 48.4%, with our model surpassing it by 16.8%, 38.2%, and 27.4%. In the daytime scenario without the daytime training dataset, SDGTrack recorded a MODA of 95%, an IDF1 of 90.8%, and a HOTA of 92.9%. In comparison, the best-performing baseline model, CSTrack, achieved a MODA of 91.1%, an IDF1 of 69.4%, and a HOTA of 79.5%, with our model outperforming by 3.9%, 21.4%, and 13.4%.

Through a comparative analysis of the test results across the three scenarios, SDGTrack demonstrated outstanding performance not only in known environments but also maintained strong performance despite environmental changes such as transitions between daytime and nighttime. Additionally, in completely unknown scenarios, SDGTrack showed significant improvements in metrics such as HOTA, MOTA, IDF1, and ID switches compared to other models, indicating superior cross-scenario generalization. This highlights its effectiveness and robustness in multi-object tracking tasks.

### 3.3. Ablation Experiment

To further validate the effectiveness of SDGTrack in complex and dynamic environments, we conducted ablation experiments. These experiments were based on our custom dataset, with the test set covering three distinct scenarios, as illustrated in Figure 1. By comparing performance across these scenarios, we aimed to better analyze the model’s tracking capabilities under varying environmental conditions. We compared the results of our model with the baseline model CSTrack across the three scenarios, representing the most significant environmental variations. The results showed that SDGTrack achieved the best performance across all scenarios, with detailed metrics provided in Table 5:

As shown in Table 5, it is evident that the application of the domain-aware attention (DAA) network module led to MOTA improvements of 10.9%, 14.8%, and 2.1% in the three scenarios, resulting in an overall increase of 9.27%. For the IDF1 metric, there was an overall improvement of 26.8% across the three scenarios. Regarding HOTA, the DAA module also brought notable improvements of 9.7%, 11.7%, and 8.0% in the corresponding scenarios, with an overall enhancement of 19.9%. These results demonstrate that the DAA module contributes to improved tracking accuracy and target identity recognition and retention. Additionally, the improvements to the Re-Byte matching scheme resulted in substantial gains across all three scenarios. Overall, incorporating Re-Byte led to a 10.0-point increase in MOTA, a 30.9-point increase in IDF1, a 20.7-point increase in HOTA, and a reduction in IDs from 447 to 54, significantly reducing confusion and errors during tracking and enhancing the continuity and consistency of tracking trajectories.

**Figure 5:**
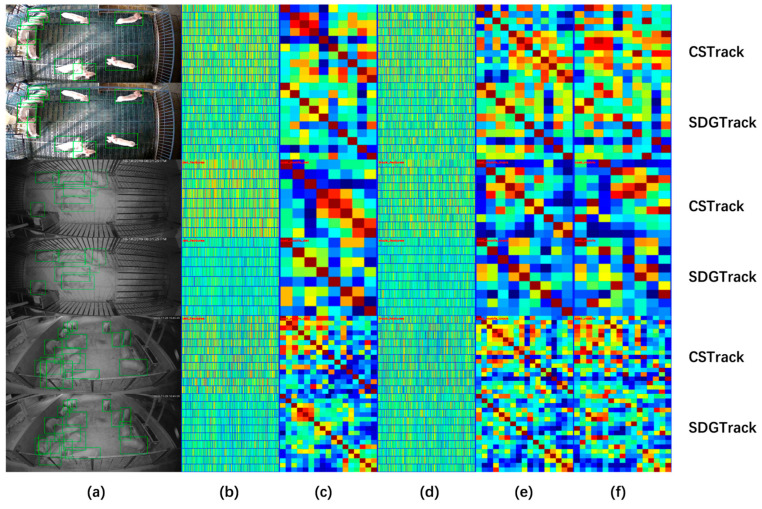
Visualizes of the discriminative capability of ID embeddings: (**a**) detection results; (**b**) visualization of ID embeddings for detected objects; (**c**) pairwise relationships between ID embeddings of detected objects in the current frame; (**d**) visualization of ID embeddings in the target sequence template; (**e**) pairwise relationships between ID embeddings in the target sequence template; (**f**) matching relationships between ID embeddings of detected objects in the current frame and those in the target sequence template.

Comparative analysis indicates that SDGTrack’s adaptive mechanisms and optimized tracking strategies provide enhanced robustness in handling complex environmental changes. Specifically, the model dynamically adjusts its detection and tracking strategies based on different scenarios, effectively reducing background interference. By integrating ID embedding features into the matching process, it improves the precision of pig identification and tracking. To visually demonstrate the improvement in ID embedding discrimination between our method and the baseline model, we conducted a visualization analysis. As shown in Figure 5, SDGTrack performed well in both the differentiation of ID embedding features and the feature association capabilities obtained through cosine similarity. Specifically, Figure 5c,d illustrate the pairwise relationships between targets, where the strong association of each target with itself is represented by a prominent dark red diagonal line in the visualization, while areas outside the diagonal appear in clear dark blue. The results indicate that incorporating embedding features into the matching process is crucial for enhancing multi-scale target detection and handling occlusions.

The experimental results demonstrate that both modules improve the model’s feature extraction and re-identification performance in unknown environments. Specifically, leveraging the DAA module enhances adaptability across different environments, effectively increasing MOTA accuracy. The Re-Byte scheme, which focuses on low-confidence targets, significantly improves the IDF1 score and reduces the number of ID switches. Our proposed SDGTrack model achieved a 16.6-point gain in MOTA, a 33.2-point gain in IDF1, a 25.2-point gain in HOTA, and reduced IDs from 447 to 24. These results indicate that SDGTrack significantly enhances tracking robustness and accuracy in new environments compared to the original model.

## 4. Discussion

Traditional tracking models perform well in specific scenarios but often suffer a sharp performance drop in unfamiliar environments. This is largely because they rely heavily on background information from specific scenarios during design and training, lacking adaptability to different environments. In pig farms, scene diversity manifests in various aspects, such as lighting conditions and environmental changes. These factors present substantial challenges in handling cross-scene tasks, particularly in different farming environments where changes in background information can significantly affect recognition and tracking. Therefore, the ability to distinguish between different backgrounds is crucial for model generalization. Our SDGTrack method addresses this issue by introducing adaptive modules and optimizing existing tracking strategies.

**Figure 6:**
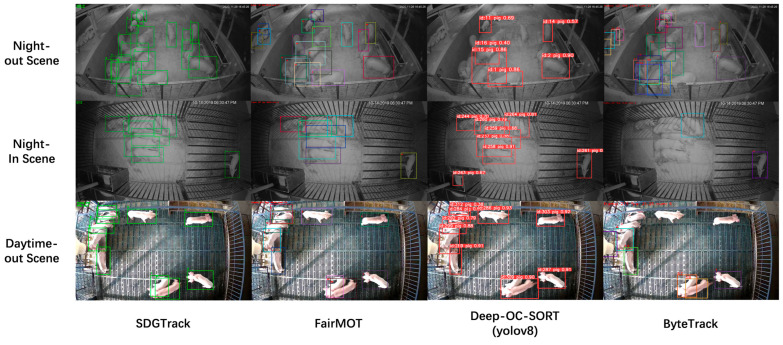
Tracking results for a typical tracking model.

We compared the tracking performance of our model with two other representative tracking solutions, including a two-stage tracking method based on the YOLO model with different tracking strategies [36,37,38,39] and the fastest anchor-free FairMOT scheme. The results, shown in Figure 6, reveal that SDGTrack not only achieved the best tracking performance across all three scenarios but also recorded the fewest ID switches.

In general, all models performed best under well-lit daytime conditions, while their performance significantly declined in the two nighttime scenarios—particularly in the Night-Out scene. This trend indicates that illumination plays a critical role in detection accuracy and identity preservation. Poor lighting at night reduces target visibility, decreases detection confidence, and increases the difficulty of feature extraction, thereby negatively affecting overall tracking performance.

Beyond lighting conditions, background complexity and pig density also had a noticeable impact on model performance. In the Daytime-Out scenario, despite similar lighting to the training data, differences in pen layout and spatial structure made background modeling more difficult, leading to degraded detection accuracy and increased identity confusion. In the Night-Out scenario, where pig density was the highest, frequent occlusions occurred, compounded by complex background textures and variable lighting, resulting in more false detections and degraded identity matching.

In contrast, SDGTrack demonstrated superior adaptability under these challenging conditions, owing to its enhanced domain-aware attention mechanism and more robust identity association strategy. These findings further confirm that designing a highly robust multi-object tracking model requires comprehensive consideration of key environmental factors, including lighting conditions, background complexity, and object density.

**Figure 7:**
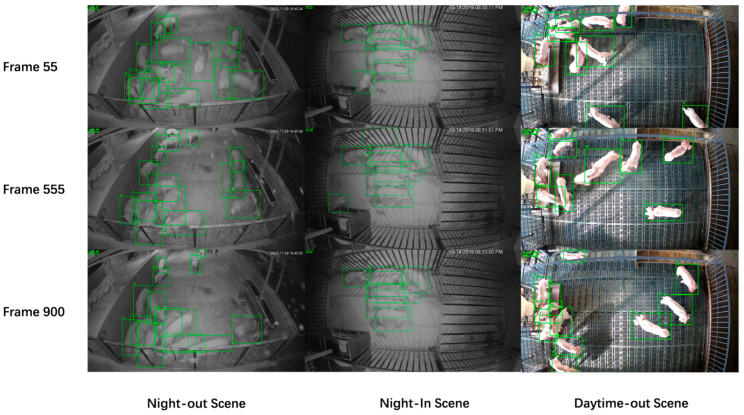
Tracking performance of SDGTrack in three scenarios.

To provide a more intuitive demonstration of the superior performance of the SDGTrack model, we present its tracking results across three different environments, as shown in Figure 7. The results clearly indicate that SDGTrack consistently maintains strong tracking performance and re-identification capabilities across various scenarios. Even in the presence of occlusions and other complex situations, the model demonstrates high accuracy. However, it is worth noting that in cases of extreme occlusion, there is still room for further optimization and improvement.

Although the SDGTrack model has made significant progress in diverse scenarios, several issues remain that need to be addressed. Firstly, in extremely complex or dynamically changing environments, the model may still experience a decline in tracking accuracy, particularly when handling large-scale occlusions or fast-moving targets. Secondly, the current model’s training and inference speed remain a challenge, especially for real-time applications. Enhancing computational efficiency and reducing latency are critical areas for future optimization. Additionally, SDGTrack currently focuses primarily on individual tracking within group-housed pig environments and has not yet addressed more complex behavior patterns or tracking tasks involving different species. The model still faces limitations in capturing more intricate social behaviors and environmental adaptability in dynamic settings. Future improvements will aim to enhance the model’s robustness in handling occlusion and motion dynamics, incorporate more advanced behavior modeling strategies, and improve real-time processing capabilities to support broader tracking applications.

It is worth emphasizing that SDGTrack not only achieves technical improvements but also demonstrates strong practical potential in handling complex and variable farming environments. In real-world pig farming scenarios, environmental factors such as lighting fluctuations, diverse pen structures, and changes in animal density can significantly impact the stability of traditional detection and tracking systems. With its robust adaptability to occlusions, lighting variations, and complex backgrounds, SDGTrack enables consistent and accurate tracking of individual pigs even under dynamically changing conditions. This capability supports more reliable health monitoring, behavioral analysis, and anomaly detection, especially in farms where environmental conditions vary frequently.

Moreover, the domain-adaptive architecture of SDGTrack provides a solid foundation for scaling to larger or multi-species farming systems. With moderate retraining or data adaptation, the framework can be extended to tracking tasks involving other livestock such as cattle or sheep. Notably, compared to sensor-based monitoring methods, SDGTrack can run efficiently on mid-range GPUs without relying on high-performance computing platforms. The system requires only standard RGB video input and does not depend on expensive sensors or dedicated hardware, significantly reducing deployment costs and minimizing direct interference with animals. These advantages offer strong economic feasibility and practical applicability, facilitating the advancement of precision livestock farming and improving both productivity and animal welfare.

## 5. Conclusions

To address the poor performance of existing tracking models in complex and dynamic farming environments, this paper proposes a novel multi-object tracking model, SDGTrack, designed to enhance generalization and tracking stability across diverse scenarios. First, the method incorporates domain-aware attention (DAA) strategies to strengthen the model’s adaptive learning capabilities for environmental information, thereby improving its generalization to unseen target domains. Additionally, by integrating our Re-Byte scheme, which combines IOU distance and ID feature distance for association, we not only optimize tracking accuracy but also significantly reduce the number of ID switches, allowing the tracking box to more precisely follow the target object. The improved model shows a marked enhancement in its ability to generalize from a single source domain to other unseen target domains. Experimental results confirm that the SDGTrack method demonstrates outstanding tracking performance across diverse farming environments, whether in bright daytime or dim nighttime scenarios. The method achieves significant improvements in key evaluation metrics, such as Multi-Object Tracking Accuracy (MOTA) and Identity F1 Score (IDF1), clearly proving its strong cross-domain generalization capabilities. Ultimately, SDGTrack provides a reliable and scalable technical foundation for real-world deployment in livestock and poultry tracking tasks under variable and challenging environmental conditions, contributing to the advancement of intelligent animal farming systems.

## Figures and Tables

**Table 1:** Composition of the constructed dataset.

Dataset	Scenario Type	Scene Count	Total Images	Description
Training	Daytime	7	12,600	Data from 7 different daytime pig life scenarios
Test	Night-In Scene	1	1800	A nighttime sequence captured in the same location as the training set.
	Night-Out Scene	1	1800	Nighttime data recorded in a farming scenario completely different from the training set.
	Daytime-Out Scene	1	1800	A daytime recording from another farm not included in the training.

**Table 2:** Evaluation metrics and terms for MOT methods.

Metric	Description
HOTA↑	Combined accuracy of detection and identity tracking.
MOTA↑	Evaluates the overall accuracy of the multi-object tracking algorithm.
IDF1↑	Combines correctly detected objects (IDTP), false positives (IDFP), and missed objects (IDFN) into a single metric.
MOTP↑	Measures the precision of the tracker in estimating the positions of targets.
MT↑	Assesses the proportion of targets that can be consistently tracked throughout the process.
ML↓	Evaluates the proportion of targets that are lost during the multi-object tracking process.
IDS↓	Represents the total number of ID switches
FP↓	False Positive (FP) refers to negative samples incorrectly predicted as positive by the model, also known as the false alarm rate.
FN↓	False Negative (FN) refers to positive samples incorrectly predicted as negative by the model, also known as the miss rate.
FPS↑	FPS represents the frame rate of the entire tracking framework.

↑ indicates performance metrics where higher values are preferred; ↓ indicates metrics where lower values are preferred.

**Table 3:** Comparative analysis of different models.

Method	HOTA↑ (%)	MOTA↑ (%)	IDF1↑ (%)	MT↑ (%)	ML↓ (%)	FP↓	FN↓	IDS↓	FPS↑
SORT (yolox)	50.8	45.4	56.8	17	9	7019	27,579	126	37.5
DeepSORT (yolox)	48.8	44.2	53.9	18	9	8693	26,650	171	12.3
Deep-OC-SORT (yolov8)	55.4	51.5	59.5	19	11	575	29,722	567	24.1
BoT-SORT (yolov8)	51.5	49.6	53.5	16	11	275	31,188	596	23.2
OC-SORT (yolov8)	55.3	51.5	59.4	18	12	575	29,720	572	20.2
ByteTrack	47.3	46.6	48.0	17	9	5498	28,128	358	37.4
FairMOT	55.1	55.6	54.6	25	5	7561	20,337	338	40.5
CSTrack	57.7	64.3	51.8	26	6	1476	20,719	447	21.6
SDGTrack (Ours)	83.0	80.9	85.1	38	3	3394	13,163	24	27.5

↑ indicates performance metrics where higher values are preferred; ↓ indicates metrics where lower values are preferred.

**Table 4:** Performance comparison of various models in different testing scenarios.

Method	Dataset	HOTA↑ (%)	MOTA↑ (%)	IDF1↑ (%)	MT↑ (%)	ML↓ (%)	FP↓	FN↓	IDS↓	FPS↑
Deep-OC-Sort (yolov8)	night-in scene	67.9	64.1	71.9	5	3	0	6436	25	15.7
	night-out scene	33.0	28.2	38.5	1	9	0	22,759	298	27.9
	daytime-out scene	83.9	90.0	78.2	13	0	575	527	244	36.0
FairMOT	night-in scene	62.5	68.8	56.8	6	1	760	4780	85	22.0
	night-out scene	39.0	33.5	45.5	7	4	6266	14,972	92	25.7
	daytime-out scene	80.0	90.5	70.7	12	0	535	585	161	40.5
CSTrack	night-in scene	56.2	64.7	48.8	5	3	1	6191	79	16.8
	night-out scene	48.4	52.8	44.4	10	4	1142	13,849	174	23.2
	daytime-out scene	79.5	91.1	69.4	11	0	333	679	194	45.6
SDGTrack	night-in scene	80.0	78.1	81.9	9	0	1558	3371	11	17.2
	night-out scene	75.8	69.6	82.6	17	2	1678	9342	10	41.3
	daytime-out scene	92.9	95.0	90.8	12	0	176	500	3	48.8

↑ indicates performance metrics where higher values are preferred; ↓ indicates metrics where lower values are preferred.

**Table 5:** Ablation analysis of the SDGTrack model.

Method	Dataset	DAA Re-Byte	HOTA↑ (%)	MOTA↑ (%)	IDF1↑ (%)	IDS↓
CSTrack	night-in scene	🗴 🗴	56.2	64.7	48.8	79
	night-out scene		48.4	52.8	44.4	174
	daytime-out scene		79.5	91.1	69.4	194
	Total		57.7	64.3	51.8	447
SDGTrack	night-in scene	✓ 🗴	75.9	75.6	76.2	90
	night-out scene		70.1	67.6	72.6	166
	daytime-out scene		87.5	93.2	82.2	208
	Total		77.6	76.7	78.6	464
	night-in scene	🗴 ✓	75.4	71.3	79.7	20
	night-out scene		68.1	62.3	74.4	29
	daytime-out scene		93.2	93.0	93.5	5
	Total		78.4	74.3	82.7	54
	night-in scene	✓ ✓	80.0	78.1	81.9	11
	night-out scene		75.8	69.6	82.6	10
	daytime-out scene		92.9	95.0	90.8	3
	Total		82.9	80.9	85.1	24

↑ indicates performance metrics where higher values are preferred; ↓ indicates metrics where lower values are preferred.

## Data Availability

Data available upon request.
